# Long-term occupational risk of latent tuberculosis infection in Hamburg, Germany: Findings from a 13-year prospective observational study

**DOI:** 10.1016/j.jctube.2025.100558

**Published:** 2025-08-13

**Authors:** Roland Diel, Matthias Gröschel, Albert Nienhaus

**Affiliations:** a Institute of Epidemiology, University Hospital Schleswig-Holstein, Kiel, Germany; b LungClinic Grosshansdorf, Germany, Airway Research Center North (ARCN), German Center for Lung Research (DZL), Germany; c Department of Infectious Diseases, Respiratory, and Critical Care Medicine, Charité – Universitätsmedizin Berlin, Germany; d German Center for Infection Research, Borstel Site, Borstel, Germany; e Institution for Statutory Accident Insurance and Prevention in the Health and Welfare Services (BGW), Hamburg, Germany; f Institute for Health Service Research in Dermatology and Nursing, University Medical Center Hamburg-Eppendorf, Hamburg, Germany

**Keywords:** Tuberculosis, Healthcare workers, Personal protective equipment, Contact tracing, Guidelines, IGRA testing

## Abstract

**Background:**

Only limited population-based data are available on the risk of latent tuberculosis infection (LTBI) in health care workers (HCWs).

**Objective:**

To assess the long-term effects of protective measures of HCWs on LTBI risk in Hamburg, Germany.

**Methods:**

Close contacts of smear-positive and smear-negative, but culture-confirmed, pulmonary TB index cases were prospectively enrolled from June 2005 to December 2017 and tested with the QuantiFERON TB (QFT) test approximately eight weeks after last exposure**.** Sociodemographic and clinical data were collected by trained healthcare personnel using a standardized questionnaire.

Contacts with known previous positive TST or IGRA results were excluded.

**Results:**

After exclusion of prevalent TB cases and contact persons who had been tested positive in other settings, valid results were available for 937 index cases and 6980 close contacts (average per case 7.45; standard deviation (SD) ± 9.99; range 1–83). Of the contacts, 3459 (49.6 %) were males and 3520 (50.4 %) females. 771 contacts (11.05 %) belonged to 11 HCW subgroups, most of them (475, or 62.8 %) hospital or geriatric nurses. Foreign-born HCW did not differ significantly from non-HCW regarding origin from high-incidence countries.

By adjusting for confounders, logistic regression analysis confirmed household contact as strongest predictor for acquiring LTBI (OR 3.8, p < 0.001), followed by foreign-born status (OR 2.2, p < 0.001) and male gender (OR 1.28, p < 0.001). Contact with a smear-positive index case only slightly increased the risk of IGRA positivity, by 16 % (OR 1.16, p = 0.024). For each additional year of age, higher odds were found at 1.86 % (OR 1.019, p < 0.001] and for each additional hour of contact at approximately 0.11 % (OR 1.011, p < 0.001). BCG vaccination had no significant effect on IGRA test results (OR 0.95, p = 0.41).

Employment in healthcare overall was associated with a 26 % lower risk of IGRA positivity compared to non-HCWs (OR 0.74, p = 0.013); however, in a second adjusted model focusing on specific HCW subgroups, this risk reduction was statistically significant only for hospital and geriatric nurses, with no significant difference observed in other HCW subgroups.

**Conclusion:**

Working in a health-care facility overall was associated with a lower LTBI risk compared to other risk factors. These findings suggest that protective measures might be particularly effective in hospital and geriatric nursing, while no risk reduction was evident for other HCW subgroups. Continued targeted protective measures remain important in high-risk care environments and support the relevance of recommendations issued (and last updated 2023) by the German Central Committee against Tuberculosis (DZK).

## 1 Introduction

In 2023, more than 10 million people fell ill with tuberculosis (TB). The annual number of cases had been falling but is in ascendance again since 2021 [1]. About a quarter of the global population is estimated to have been infected with TB [2]. In low-incidence countries, employees in healthcare facilities who have unprotected contact with infectious TB patients are at considerable risk of *Mycobacterium tuberculosis* (MTB) transmission [3,4].

Current guidelines, such as those in the U.S, the UK and Germany, recommend a bundle of complementary infection control measures to reduce such transmission [5,6,7]. Intermittent training sessions for medical personnel are conducted by infection control officers at individual Hamburg (Germany) hospitals and other healthcare facilities. In addition, the Institution for Statutory Accident Insurance and Prevention in the Health and Welfare Services (BGW) and Public Health Service offer expertise on the risks posed by undiagnosed TB patients and emphasise the proper use of personal protective equipment (PPE). Attempts have not yet been carried out to evaluate the overall effectiveness of these recommendations under routine long-term conditions. Although routine PPE use is a key matter brought up by Public Health personnel conducting the contact investigations that are required when patients are diagnosed with infectious pulmonary TB, evidence suggests that retrospective employee surveys on personal protective measures against infections can be influenced by memory gaps or biases. Factors such as the time elapsed between the event and the survey, the complexity of the information being sought, and individual differences in recall can affect the accuracy of responses [8,9].

More objective, albeit indirect, information on the effectiveness of protective measures for healthcare employees against MTB infection under real-world conditions can be obtained from long-term, population-based reviews of contact investigations that include well-characterized contacts and their index cases. One such study was initiated in Hamburg, Germany, which was the first city in Germany to introduce routine Interferon-Gamma Release Assay (IGRA) testing in Public Health services in 2005 with positive results as surrogate evidence of latent TB infection (LTBI). Since then, all tested close contacts of patients with infectious pulmonary TB (so called “index patients”, i.e., the source case from which the contact investigation originated) have been systematically recorded. IGRAs offer several advantages over the tuberculin skin test (TST), particularly improved specificity in BCG-vaccinated individuals and the convenience of requiring only a single visit for blood collection and in vitro processing [10].

The site of the present study, Hamburg, is one of Germany’s federal states and, with nearly 2 million residents (1.946 million as of December 31, 2022 [11], the country’s second-largest city. Hamburg has the highest TB incidence rate among all 16 federal states, with 10.8 cases per 100,000 inhabitants in 2023 [12]. Coincidentally, it also has the second-highest proportion of foreign-born residents (20.7 %), compared to the German national average of 15.2 %, as of December 31, 2023 [13].

In the present analysis, we report the results of LTBI testing as part of contact tracing for all close contacts between 2005 and 2017, i.e., over a 13-year period. We specifically examine HCWs and non-healthcare workers and compare the factors influencing the acquisition of latent tuberculosis infection between these two groups.

## 2 Material and methods

### 2.1 Definition of HCWs

HCWs were defined as all medical, dental, nursing, obstetric or assisting persons in different areas, e.g. hospitals, outpatient clinics, doctors' surgeries practices, dialysis facilities, nursing homes and out-patient care facilities.

### 2.2 Legal requirements and inclusion criteria

The contact tracing study is embedded in the mandatory routine surveillance conducted by the Public Health Department, as legally mandated by the German Infectious Diseases Law (IfSG) and has been approved by the Hamburg Commissioner for Data Protection. It includes all patients with culture-confirmed pulmonary TB who were residents of Hamburg and, therefore, had to be reported by name in accordance with paragraph 6 of the German Infectious Diseases Law (IfSG). Furthermore, the IfSG (paragraph 16, 25 and 34) explicitly references and mandates contact investigations for TB to control and prevent its spread within the community. The German Central Committee against Tuberculosis (DZK) provides detailed guidelines for conducting TB contact investigations [14]. To increase the likelihood of detecting MTB infections the guidelines call for carefully selecting individuals for IGRA or TST testing, focussing on “close” contacts only. A positive IGRA or TST leads in close contacts results in a diagnosis of latent TB infection (LTBI). According to these recommendations, a contact is classified as “close” if there was a cumulative exposure period of at least 8 h to a sputum smear-positive or at least 40 h to a smear-negative but culture-confirmed pulmonary index patient in enclosed spaces. Alternatively, brief face-to-face exposure to direct coughing by a sputum smear-positive patient was also considered “close.” Testing only 'casual' contacts with a low pre-test probability of recent MTB infection may reduce the positive predictive value of an LTBI test, thereby increasing the likelihood of false-positive results. Accordingly, contact persons who did not meet these specific criteria were not tested by the Public Health department and therefore not included in our study. Contacts who had previously developed active TB but were only incidentally diagnosed by chest X-ray following a positive IGRA result during contact investigation (i.e., prevalent TB cases) were also excluded.

### 2.3 Questionnaire

To ensure proper identification of close contacts, sociodemographic and clinical data were collected as a matter of routine during interviews of supposed close contacts. These were conducted by trained public health staff using a standardized questionnaire. With respect to index persons, the time lag between initial presentation and definitive diagnosis was collected to identify the period during which MTB transmission could have occurred, and the laboratory results of microbiological investigations were recorded to determine whether the index persons were smear-positive or only culture-positive but smear-negative.

The following information was obtained from the survey of each contact: gender, age, country of birth, whether the contact was a household contact or not, the type of employment of the patient (particularly regarding healthcare activities), the time elapsed since the last possible exposure date, whether they had previously been BCG-vaccinated, whether they had ever been tested for LTBI in the context of a previous investigation and whether they had previously fallen ill with TB in their lifetime. When a contact is identified as a healthcare worker, additional interviews are conducted regarding the type of medical facility and the nature of TB exposure risks in that setting.

Contact exposure time was determined according to the recommendations of the German Central Committee against Tuberculosis [14] by interviewing both the contact person and, in case of uncertainty, their respective index case.

Contact time was recorded in six consecutive 4–hour blocks per day, with only time exceeding each full block counted toward total exposure; shorter contacts were documented narratively. This method has been published previously [15] and also applies to household contacts, where prolonged exposure periods can often be found [16,17].

### 2.4 IGRA testing

All included contacts, with exception of children aged below 5 years for whom the TST is usually preferred, were tested with the QuantiFERON-TB Gold In-Tube test (QFT-GIT) since 2005 or, since 2016, with the QuantiFERON-TB Gold Plus test (QFT-Plus). Referred to together simply as QFT, these are products of QIAGEN, Hilden, Germany. Blood samples were collected at least 8 weeks following the contact person’s last possible exposure to the respective index case. According to the manufacturer's guidelines, a QuantiFERON-TB Gold Plus (QFT-Plus) test is considered positive if the IFN-γ response in at least one of the TB antigen tubes exceeds 0.35 IU/mL after subtraction of the Nil value.

### 2.5 Statistical analysis

Before performing multivariable logistic regression, univariable analyses were conducted to identify potential predictors of IGRA positivity and assess differences between HCWs and non-HCWs. Categorical data were compared by the Pearson’s x^2^ test (or Fisher’s exact test, when expected sample sizes comprised fewer than five subjects). The Wilcoxon rank sum test was performed to determine whether the distribution of continuous variables differed between two groups.

Independent predictors of the primary outcomes were identified through logistic regression models, adjusting for relevant confounders. Variables considered relevant for inclusion were age, sex, country of origin (German-born or foreign-born), history of BCG vaccination (determined by vaccination record or presence of a scar), employment as healthcare worker, household contact (as a special subgroup of non-HCWs), exposure time of the contacts to their source cases, and sputum-smear positivity of the corresponding index case. Relations are expressed as odds ratios (OR) and 95 % confidence intervals (CI) for each risk factor, with significance assessed by P values computed from Wald statistics. All P values reported are based on two-tailed comparisons with statistical significance set at P < 0.05.

In addition, a classification tree analysis was conducted to identify subgroups with particularly high or low IGRA positivity rates. The tree was generated using the ‘DecisionTreeClassifier‘ from the ‘sklearn‘ library in Python, with IGRA positivity as the binary outcome. Predictors included age, gender, foreign-born status, BCG vaccination, healthcare worker status, household/intimate contact, exposure time, and index case smear status. In a secondary analysis restricted to HCWs, the tree excluded household contact as a predictor. Trees were visualised with node IDs displayed using ‘plot_tree()‘, and key nodes with high IGRA positivity were manually highlighted.All statistical analyses were performed using SPSS version 29.0.2.0 (IBM Corp., Armonk, NY), JASP version 0.19.3 (University of Amsterdam), and Python 3.11 with the scikit-learn library version 1.4.1.

## 3 Results

### 3.1 Overall characteristics of index persons and their contacts

As of 31 December 2017, a total of 7014 contact persons could be identified who met the definition of a “close” contact person, as defined by the current DZK recommendations. Contacts with a known positive TST or IGRA result had not been re-tested for LTBI and were therefore not included in our study.

Of the 7,014 contacts initially identified, 31 prevalent TB cases—i.e., individuals who had already developed active TB by the time of contact tracing and for whom transmission could not reliably be attributed to the presumed index case—were excluded a priori. Furthermore, the QFT test yielded an indeterminate result in three contact persons (0.32 %) in whom no medical cause could be identified upon closer examination. These individuals were also excluded from further analysis. Thus, 6,980 contacts linked to one of 937 index cases remained. 580 (61.9 %) of the 937 index cases were sputum smear-positive and 357 (38.1 %) were sputum smear-negative.

Males made for 3461 (49.6 %) of all 6980 contacts and females for 3519 (50.4); the sex ratio was thus about 0.98 to 1. This gender relationship did not differ for the 3101 foreign-born (44.4 % of all 6980 contacts), 1688 (54.4 %) of whom were male and 1413 female (45.6 %). The contacts were on average aged 35.4 years (standard deviation [SD] ± 13.6). On average, each of the 937 patients with culture confirmed and thus potentially infectious pulmonary TB had on average 7.45 contact individuals (SD ± 9.9; range, 1–83). The number of contact persons investigated did not differ significantly (p = 0.13) between German-born (total, 3879; average 8.0; SD ± 10.8) and the foreign- born (total, 3101; average 6.0; SD ± 8.9). Our subjects included 273 children aged below 15 years of age during the study period, with a mean age of 11.1 (SD ± 2.62 years), of whom 198 (72.5 %) were born in Germany. Immunosuppressive therapy, haematological malignancies or other severe underlying diseases which might have compromised the QFT test due to impaired T-cell responses, potentially resulting in false-negative or indeterminate results, were not reported.

### 3.2 Characteristics of healthcare workers

Of the 6980 contacts, 771, or 11.05 % were HCWs. An overview of key characteristics of the study population – including age, sex, foreign-born status, contact time, and household exposure – is provided in Table 1. Of those, 485 (62.8 %) were hospital or geriatric nurses, 24 (3.1 %) private physicians, 145 (18.8 %), nurses in private practice, 9 (1.2 %) hospital doctors, 7 (0.9 %) dentists, 41 (5.3 %) dental assistants, 9 (1.2 %) cleaners, 9 (1.2 %) physio-therapists, 35 (4.5 %) paramedics or ambulatory drivers, 6 (0.8 %) employees in a pharmacy and 2 (0.2 %) midwifes. Among HCWs, only slightly more than one quarter (28.7 %) of exposed contacts were men. HCWs were on average 4 years older 39.0 (SD ± 11.0) vs 35.0 (SD ± 13.9) years, p < 0.001) than other contact persons. Of the 3,101 contact persons (44.6 %) who were born outside Germany, a total of 127 countries of origin were represented, and none originated from any of the 30 “high-burden countries” as defined by the WHO [1]. Just 11 countries accounted for nearly two-thirds (65.6 %) of all foreign-born individuals: 12.8 % (398/3,101) were born in Afghanistan, 12.6 % (392/3,101) in Turkey, 8.4 % (260/3,101) in Russia, 7.8 % (242/3,101) in Poland, 6.7 % (207/3,101) in Kazakhstan, 5.5 % (171/3,101) in Eritrea, 3.5 % (108/3,101) in Syria, 2.7 % (83/3,101) in Iran, 2.3 % (70/3,101) in Vietnam, 1.8 % (57/3,101) in Kosovo, and 1.7 % (54/3,101) in Romania.

**Table 1:** Univariate analysis of risk factors for latent TB separated by HCWs and Non-HCWs.

Characteristic	HCW group (N = 771)	Non-HCW contacts (N = 6209)	All contacts (N = 6980)	p-value
Age (yr) mean ± SD	39.1 ± 11.0	35.0 ± 13.9	35.4 ± 13,6	<0.001
Foreign-born (n, %)	230/771 (29.8)	2871/6209 (46.2)	3101/6980 (44.4)	<0.001
Male (n, %)	220/771 (28.5)	3241/6209 (52.2)	3461/6980 (49.6)	<0.001
Sputum smear positivity of source case (n, %)	453/771 (58.8)	3923/6209 (63.2)	4376/6980 (62.7)	<0.02
Previous history of TB (n, %)	2/771 (0.3)	18/6209 (0.3)	20/6980 (0.3)	ns
BCG vaccination (n, %)	328/771 (42.5)	2034/6209 (32.8)	2353/6980 (33.8)	<0.001
Cumulated exposure time (h) ± SD	30.47 (95.63)	102.60 (198.49)	94.64 (191.23)	<0.001
Cumulated exposure time < 1h (n, %)	122 (15.8 %)	144 (2.3 %)	266 (3.8 %)	<0.001
Cumulated exposure time ≥ 8h (n, %)	330 (42.8 %)	4948 (79.7 %)	5278 (75.6 %)	<0.001
Cumulated exposure time ≥ 40 h (n, %)	129 (16.7 %)	3069 (49.4 %)	3198 (45.8 %)	<0.001
QFT positivity (n, %)	88 (11.4 %)	1338 (21.5 %)	1426 (20.4 %)	<0.001
QFT positivity in sputum smear positives (n, %)	50 (11.0 %)	852 (21.7 %)	902 (20.6 %)	<0.001
QFT positivity when exposed < 1 h (n, %)	10 (8.2 %)	22 (15.3 %)	32 (12.0 %)	<0.001
QFT positivity when exposed ≥ 8h (n, %)	47 (14.2 %)	1164 (23.5 %)	1211 (22.9 %)	<0.001
QFT positivity when exposed ≥ 40 h (n, %)	17 (13.2 %)	897 (29.2 %)	914 (28.6 %)	<0.001

Among foreign-born HCWs (n = 230), 106 individuals (46.1 %) originated from countries with a TB incidence ≥ 40 per 100,000 population, compared with 1,395 of 2,871 foreign-born non-HCWs (48.6 %). This difference was not statistically significant (two-proportion z-test: z = –0.73, p = 0.46; chi-square test: χ^2^(1) = 0.53, p = 0.47). Thus, while the overall proportion of foreign-born individuals was lower in the HCW group (29.8 % vs. 46.4 %), the distribution of origins from high-incidence countries among the foreign-born was comparable between HCWs and non-HCWs.

As could be expected from the strict criteria for including contact persons in IGRA testing, 75.6 % of contacts had a cumulative exposure time ≥ 8 h: and 45,8% ≥ 40 h. Only 266, or 3.8 %, of contacts had an exposure time < 1 h. A substantial proportion (45.9 %) of those short-exposure contacts were HCWs (122/266), making up 15.8 % of all HCWs contacts. On average, cumulative exposure time in HCWs was significantly lower with 30.5; SD ± 95.6 h versus 102.6; SD ± 198.5 h in non-HCWs (p < 0.001).

### 3.3 Risk factors for acquiring latent TB (univariate analysis)

Our work began with the performance of univariate analysis to identify significant differences in IGRA positivity between the 771 HCWs and the 6209 other contacts (see Table 1) considering key influencing factors. Additionally, the analysis explores the association between IGRA positivity and selected factors themselves.

a) Impact of foreign-born status

IGRA positivity in foreign-born contacts was significantly higher than in German-born (883/3101, or 28.5 % vs. 543/3879, or 14.0 %, p < 0.001). This supports the hypothesis that a positive QFT result may often be due to earlier-life exposure to infectious TB in the high-TB-burden home country, rather than recent infection from the suspected index case. Nevertheless, foreign-born HCWs had a significantly lower IGRA positivity rate (19.13 %, 44/230) compared to foreign-born non-HCWs (29.22 %, 839/2,871), with a statistically significant difference (p = 0.0011), suggesting that the safety measures they used in the case at hand provided effective protection that their non-HCWs counterparts did not have.

b) Impact of sputum-smear positivity

Overall, in 4376, or 62.7 %, of all contacts the index cases were sputum-smear-positive. Of the HCWs exposed to sputum-positive index cases, only 50 out of 453 (11.04 %) tested IGRA-positive, whereas among those exposed to sputum-negative index cases, 38 out of 318 (11.95 %) were IGRA-positive. The difference was not statistically significant (p = 0.695), indicating that sputum-smear status of the index case did not significantly impact IGRA positivity among HCWs. With respect to household contacts, the IGRA-positivity rate was significantly higher compared to non-household contacts, irrespective of the sputum-smear status of index cases (49.31 % vs. 16.83 % and 30.95 % vs. 16.55 %, respectively, each p < 0.001). These findings indicate that household exposure significantly increases the likelihood of IGRA positivity, regardless of the index case smear status.

c) Impact of BCG-vaccination

BCG-vaccination was significantly more common among foreign-born contacts, likely reflecting the national vaccination policies in their home countries: Of German-born contacts, 1218/3879 (31.4 %) were BCG-vaccinated, vs 1135/3101 (36.6 %) of the foreign-born contacts (p < 0.001).

BCG vaccination appeared not to influence the QFT results. Among the BCG-vaccinated, 464/2353 (19.7 %) were QFT-positive. The positivity rate was practically the same for the 962/4626 unvaccinated contacts (20.8 %), meaning that no statistically significant difference in IGRA positivity was found between the vaccinated and the unvaccinated (p = 0.31). Taking foreign-born contacts alone, there was again a nearly identical IGRA positivity rate between BCG-vaccinated (319/1135, or 28.1 %) and unvaccinated individuals 564/1966, or 28.7 %). Interestingly, BCG-vaccinated HCWs had a slightly higher IGRA positivity rate (41/320, or 12.8 %) than non-vaccinated HCWs (47/451, or 10.4 %), but this difference is not statistically meaningful. At first glance, these results suggest that BCG vaccination does not provide strong protection against IGRA positivity in our cohort.

d) Impact of being a household contact

Household contacts had a 2.7 times higher IGRA positivity rate (410 out of 907; 45.20 %) compared to non-household contacts (1,016 out of 6,073; 16.73 %, p < 0.001). Among the 3101 foreign-born contacts, IGRA positivity was 53.8 % (281/522) for household contacts compared to 23.3 % (602/2579) for non-household contacts (p < 0.001). This corresponds to a 2.31–fold higher IGRA positivity also in foreign-born household contacts, which is even more pronounced than in the overall cohort (2.7–fold; 45.2 % vs. 16.7 %, p < 0.001). This emphasises the strong influence of close and prolonged exposure in household settings on the risk of TB transmission.

e) Impact of cumulative exposure time

The cumulative exposure time among QFT-positive contacts was on average 58 h longer than among those who were QFT-negative (mean 140.7; SD ± 196.7 vs. 82.0; SD ± 187.99 h, p < 0.001). Also, HCWs who were IGRA-positive had a slightly longer average exposure time than QFT-negative HCWs (36.69; SD ± 120.62 vs. 29.67; SD ± 91.99 h), but this difference was not statistically significant (p = 0.091). This suggests that exposure time alone may not be the key factor influencing IGRA positivity in HCWs, and other variables (e.g., protective measures, job role, prior exposure) may play more significant roles.

f) Impact of employment as HCWs

Only 88 out of the 771 HCWs (11.4 %) tested were IGRA-positive and thus only about half as likely to be IGRA-positive than other contact persons (1338/6029, or 21.5 %).

Nurses (57/485, or 11.75 %) and medical assistants in private practices (13/145, or 8.97 %) had significantly lower IGRA positivity rates compared to contacts outside their respective occupational group (21.08 % and 20.67 %, each p < 0.001), suggesting that specific healthcare professions may be associated with a lower TB exposure risk. The other 18 HCWs who tested QFT-positive were distributed across the other HCWs subgroups, the low IGRA positivity rates of which did not differ significantly from one another.

When comparing nurses in private practice and nurses in hospital or geriatric settings, it was noticeable that nurses in private practice had significantly shorter contact times with index cases (22.9; SD ± 98.26 h vs. 36.19; SD ± 98.26 h, p < 0.001).

g) Impact of prior TB disease

Interviews revealed that prior TB disease was extremely rare among all contacts and occurred with equal frequency in HCWs and non-HCWs (0.3 % in both groups; 2 and 18 cases, respectively). Among all 6,980 contacts, only 20 individuals (0.3 %) reported a previous history of TB disease. Of these, 13 (65 %) were German-born and 7 (35 %) foreign-born, resulting in similarly low prevalence rates of 0.3 % in German-born and 0.2 % in foreign-born individuals. A chi-square test (without continuity correction) showed no significant difference in prior TB history between German-born and foreign-born contacts (χ^2^(1) = 0.39, p = 0.53).

### 3.4 Results of classification tree analysis

**Fig. 1:**
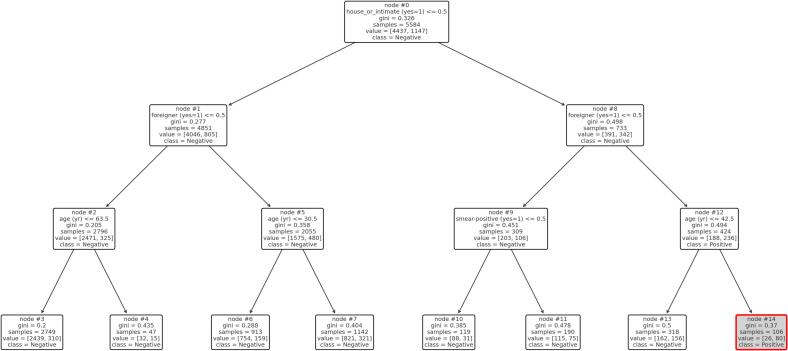
**Decision tree analysis: All contact persons.** Node 14 is highlighted in light grey with a red border. This node showed the highest IGRA positivity rate among all subgroups identified in the tree: foreign-born individuals with household or intimate contact to an index case and aged over 42.5 years.

To explore combinations of exposure-related variables associated with IGRA positivity, a classification tree analysis was conducted. The tree revealed distinct subgroups with markedly different positivity rates. In the full dataset, the highest IGRA positivity was observed in node 14 (Fig. 1), comprising 106 foreign-born individuals with household or intimate contact to an index case and aged over 42.5 years; 75 % of this group tested IGRA-positive. These findings indicate a markedly increased infection risk among older foreign-born individuals with close (household or intimate) contact to the index case.

**Fig. 2:**
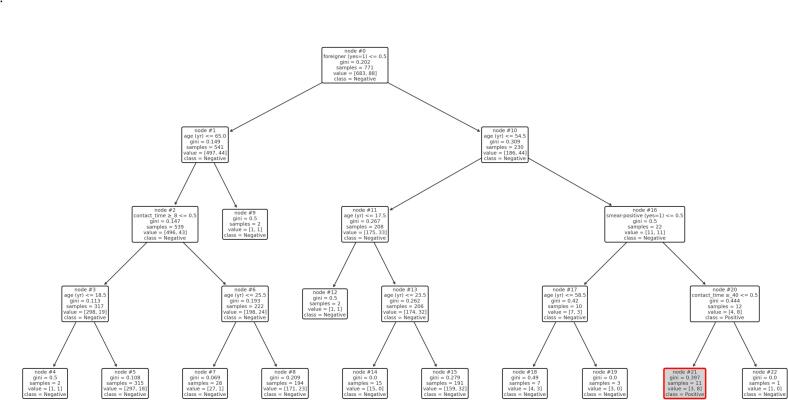
Decision tree analysis: HCWs only. Node 21 identified a particularly high-risk subgroup of HCWs: foreign-born individuals over the age of 54 who had contact with a sputum-positive index case for less than 40 h.

In contrast, the lowest positivity rate (0 %) was found among young foreign-born HCWs aged 17.5–23.5 years, in a subgroup identified by a separate tree restricted to HCWs. Among HCWs, node 21 (Fig. 2) was highlighted as the highest-risk group, consisting of older foreign-born individuals (age > 54.5) who had contact with a sputum smear-positive index case for less than 40 h; 72.7 % tested IGRA-positive in this group. Despite the relatively short exposure time, this group showed a striking IGRA positivity rate of 72.7 %, suggesting a disproportionately elevated infection risk driven by the combination of age, origin, and exposure to highly infectious cases.

### 3.5 Results of logistic regressions

Independent risk factors for recent LTBI as measured by QFT were determined by logistic regression (see Table 2). Higher Age, origin outside Germany, male gender, longer contact duration, being a household contact and sputum smear-positivity were all associated with IGRA positivity, however, to different degrees. BCG vaccination had no significant effect on IGRA test results. (OR 0.95, p = 0.41).

**Table 2:** Results of multiple logistic regression of risk factors for latent TB infection.

a) Baseline results				
Variable	OR (Odds Ratio)	Lower 95 % CI	Upper 95 % CI	P-value
const	0.052	0.041	0.066	<0.001
HCW	0.737	0.580	0.938	0.013
Foreign-born	2.228	1.964	2.527	<0.001
Age	1.019	1.014	1.023	<0.001
Gender (male)	1.284	1.133	1.455	<0.001
BCG vaccination	0.946	0.830	1.079	0.410
Household contact	3.812	3.259	4.460	<0.001
Sputum smear positivity	1.160	1.020	1.320	0.024
Cumulative contact time (h)	1.0011	1.0010	1.0013	<0.001

b) Logistic regression results including hospital or geriatric nurses and nurses in practice				
Variable	OR (Odds Ratio)	Lower 95% CI	Upper 95% CI	p-value
const	0.052	0.041	0.066	<0.001
Hospital or geriatric nurses	0.728	0.544	0.975	0.033
Nurses in practice	0.670	0.374	1.199	0.178
Foreign-born	2.232	1.968	2.532	<0.001
Age	1.019	1.014	1.023	<0.001
Gender (male)	1.283	1.133	1.454	<0.001
BCG vaccination	0.945	0.829	1.076	0.392
Household member	3.823	3.269	4.471	<0.001
Sputum smear positivity	1.161	1.020	1.321	0.024
Cumulative contact time (h)	1.001	1.001	1.001	<0.001

BCG: Bacillus Calmette-Guérin; CT: confidence interval; HCW: healthcare worker; h: hour(s); ns: not significant; QFT: Quantiferon test; yr: year.

The strongest predictor of LTBI was exposure as a household member. Subjects in this group had a nearly 4-fold higher risk of IGRA positivity (OR ratio 3.8, 95 % CI 3.3–4.5, p < 0.001), followed by foreign-born individuals, (double the risk of the German-born (OR 2.23, 95 % CI 1.96–2.53, p < 0.001). Male gender had a 28 % higher risk of IGRA positivity than women (OR 1.28, p < 0.001). Contact with a smear-positive index case brought only slightly higher risk (16 %) of IGRA positivity, suggesting that smear-negative TB cases can still be infectious (OR 0.16, p = 0.024).

Higher age was also associated with a higher risk of IGRA positivity (OR 1.0186, 95 % CI 1.014–1.023, p < 0.001). That means that for each additional year of age, the odds of IGRA positivity increase by 1.86 % corresponding to a 20.0 % increase in the odds of IGRA positivity over a decade.

Each additional hour of contact increased the odds of IGRA positivity by approximately 0.1 % (OR 1.0011, p < 0.001). The risk of IGRA positivity doubled after approximately 630 h of cumulative contact time, e.g. 26 days of continuous exposure for 24 h/day. This confirms that long-term exposure is a significant risk factor for TB transmission, even when the per-hour risk increase is small. It also underscores the importance of often prolonged household contact, particularly in cases of yet-undetected or undiagnosed pulmonary TB.

**Fig. 3:**
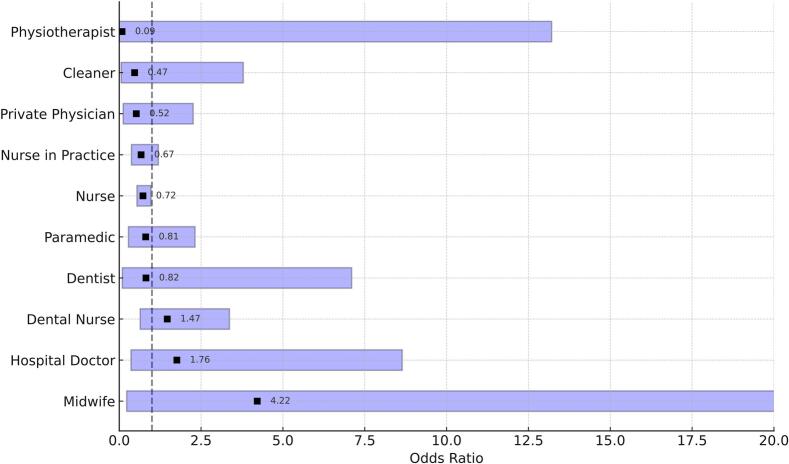
**Odds Ratios of IGRA positivity among HCW-subgroups.** Squares represent the respective odds ratios. The dashed line indicates an odds ratio of 1. The ends of the bars show the upper and lower bounds of the 95% confidence interval for the respective odds ratios.

While all previously mentioned variables increased the risk of acquiring LTBI, employment in the healthcare sector resulted in a 26 % lower risk of IGRA positivity compared to non-HCWs (OR 0.74, p = 0.013). If one replaced “HCWs”, however, as a general category in the baseline model with the individual HCW subgroups, and considered their odds separately as individual groups, only hospital or geriatric nurses remained significantly less likely to be IGRA positive (OR 0.728, p = 0.033, see Table 2). Nurses in private practice, who showed a significantly lower LTBI risk in univariate analysis, lost their significance in the logistic regression (OR 0.67, p = 0.178), suggesting that the comparatively low exposure time in this group confounded the association and was accounted for in the adjusted model. In the remaining HCW subgroups, the odds of IGRA positivity were not significant as their wide confidence intervals extended well beyond 1 (Fig. 3). This lack of significance was probably due to low numbers of individuals in these subgroups, resulting in insufficient statistical power.

The other factors (foreign-born status, household exposure, contact time, etc.) had similar effects in both models.

## 4 Discussion

There is consensus in all international guidelines that hospital staff and those visiting patients suspected of having infectious TB, or where there is a risk that a patient might be suffering from TB disease, are to wear respiratory protection. For the USA, these must satisfy or exceed the N95 standards, providing a filtration efficiency of 95 %, set by the Centers for Disease Control and Prevention (CDC) and the National Institute for Occupational Safety and Health (OSHA). For the UK and Germany, the nearly equivalent filtering face piece (FFP)2 standard with a filtration efficiency of at least 92 % and that is European Conformity (CE) certified [[5], [6], [7]] is required. This is particularly important as the risk of TB among HCWs is consistently higher than in the general population worldwide: Baussanós review [15] found the stratified pooled annual latent infection estimates even for countries with low (<10 cases/100,000 population) TB incidence to be 3.8 % (95 % CI 3.0–4.6). This emphasises the need for adequate TB infection control measures to be implemented in all health care facilities treating patients suspected of having infectious TB. Preventing MTB infections is of great importance, as curing it is difficult. Adherence to preventive chemotherapy following an LTBI diagnosis among HCWs is generally considered to be suboptimal. The literature shows that—regardless of the chosen screening method (TST or IGRA)—fewer than 50 % of cases worldwide result in proper treatment completion [[18], [19], [20], [21], [22]].

Only a few, retrospective, single-centre studies have demonstrated that the implementation of protective measures in hospitals can lead to a reduction in TB. However, these single-centre studies typically cover only short timeframes and are not representative of HCWs practices across all healthcare environments in a major metropolitan area [23,24,25]. Our study helps to close this gap. It summarises the results of standardised contact investigations involving well-characterised TB patients, and a body of close contacts representative of the general population. As concerns HCW and their inherent professional risk of contracting TB, our study provides a comparison of different risk factors and makes possible an assessment of the effectiveness over the long term of the protective measures in place in our metropole.

In our study, household contact represented the strongest independent risk factor for LTBI, despite its applying to only 12.99 % (907 out of 6,980 individuals) of all contacts. Counting accumulated hours of contact with index patients prior to their diagnosis with infectious pulmonary TB, that of household contacts was significantly longer than for others, on average 156.63 h; SD ± 175.27, compared to 85.38 h average; SD ± 191.80, for non-household contacts (p < 0.001). However, the influence of contact time alone—and thus the per-hour effect of contact time—on LTBI risk is limited, as each additional hour of contact increases the odds of IGRA positivity by only approximately 0.11 %. Therefore, the fact that household contacts had nearly four times higher odds of a positive IGRA test compared to non-household contacts is likely due not only to the longer contact duration but also to the greater intensity of contact within the familial environment. The significantly higher risk of IGRA positivity in this group, as compared to non-household contacts, holds regardless of the sputum smear status of the index case. Notably, the higher risk remains significant in both smear-positive and smear-negative index cases. Particularly when exposure periods are uncertain, priority should be given to identifying household or close relationship contacts to detect LTBI at an early stage.

The impact of increasing age on LTBI risk, as well as being born outside of Germany, was expected, as age is associated with a greater likelihood of previous undiagnosed exposures to infectious TB patients, and foreign-born individuals are often exposed earlier in life in high-prevalence countries [26]. Male sex was likewise an expected independent risk factor for IGRA positivity, as men are generally considered more susceptible to LTBI than women [27,28]. The effect of smear positivity as an independent predictor was statistically significant but weaker than expected, suggesting that smear-negative TB cases can still be infectious. This result supports the core principle of the German recommendations, which advise not to focus solely on sputum positivity, but rather to treat every culture-positive pulmonary TB case as a basis for a thorough contact investigation.

In clear contrast to the significantly increased LTBI risk among household contacts, a central finding of this study is the significantly lower LTBI risk among HCWs. Indeed, the average cumulative exposure time for HCWs was significantly lower than that for non-HCWs—30.2 h versus 101.2 h—with brief exposure of less than one hour occurring nearly seven times more frequently among HCWs (15.9 % vs. 2.3 %, p < 0.001). These lower exposure times, however, do not explain the lower infection rate among HCWs. A closer look at the exposure times reveals further differences between HCWs and non-HCWs as contact persons. When the two groups are compared in contact time-stratified groups, (cumulative contact times of less than 1 h, of at least 8 or at least 40 h, see Table 1), the HCWs have significantly lower infection rates across the groups. The lower IGRA positivity among HCWs is further supported by the observation that even among those exposed to smear-positive index cases, the IGRA positivity rate was nearly half that of non-HCWs (11.2 % vs. 21.7 %, p < 0.001).

In univariate subgroup analysis a protective effect—i.e., a significantly lower rate of IGRA positivity compared to other contacts—was statistically demonstrable only among hospital or geriatric nurses and nurses in private practice Although, the proportion of HCWs among all potential contacts (11.05 %) in our population-based Hamburg study is relatively high (compared to the general German population, where healthcare and social service workers account for only about 7 % of the total population (5.8 million of 83 million inhabitants [29]) the statistical power within HCW subgroups may have been limited. This is likely due to the distribution of the 771 HCWs across 11 distinct occupational subgroups many of which included only a small number of individuals. However, in the logistic regression model, working as a nurse in private practice was no longer an independent risk factor (see Table 2), likely due to their shorter exposure times to TB patients in outpatient settings.

Our study has some limitations. First, our study does not provide a fully comprehensive picture of the effectiveness of TB prevention measures for HCWs in Hamburg. The number of unidentified, occupationally acquired LTBI cases may be higher than reported, as only HCWs residing in Hamburg were included in our investigation. HCWs working in Hamburg but residing in surrounding districts were indeed investigated by local Public Health offices but were not part of this survey. Second, despite comprehensive and timely interviews of contacts regarding their cumulative exposure times to index cases, some degree of uncertainty due to potential recall bias must always be considered. However, such bias is likely to occur less frequently among HCWs because their specific activities (e.g., bronchoscopy, emergency department encounters, ambulance transport, outpatient nursing care) are often well documented and/or easy to recall.

Furthermore, the study does not provide data on the degree of implementation fidelity of protective measures across different healthcare settings. For example, it remains unknown whether certain HCWs consistently applied protective measures such as FFP2 masks or whether mask usage varied depending on perceived patient risk. Also, the study does not account for the possibility of HCWs acquiring LTBI from sources outside of their professional environment, which may confound the results. Additionally, while the long-term nature of the data collection adds strength to the findings, it also means that changes in screening test versions (e.g., switch from QFT-GIT to QFT-Plus in 2016) might have slightly affected IGRA positivity rates in the last 2 years of our study period. However, concordance between the QFT methods has been reported to be strong at 96.08 % [30] indicating that any resulting uncertainty is likely negligible and unlikely to have affected the overall findings.

Finally, we cannot prove cause-and-effect with our findings. Since the publication of the DZK guidelines in 1996, HCW in Germany have been trained to use FFP2 respiratory masks when sharing space with a patient suspected of having pulmonary TB. Our data indicate that those guidelines and that training have been relatively effective. They are, however, only indirect conclusions and not the results of a randomized interventional trial. While such an approach—creating two classes of HCW, one using PPE and one not, would be ethically unacceptable, retrospective interviews of HCWs regarding the use of protective measures during exposure to undiagnosed TB patients in their work environments would be supposedly prone to bias.

Thus, continuous insight into the effectiveness of control measures in healthcare facilities can only be provided by ongoing evaluation of the results of IGRA testing of close contacts in the community. This should be established as a national standard by Public Health authorities to support the fight against TB in Germany.

## Authors contribution

Roland Diel: Conception and design of the study, acquisition, statistical analysis and interpretation of data, drafting and revising of the article.

Matthias Gröschel: Interpretation of data, revising of the article, giving final approval to the version to be published.

Albert Nienhaus: Interpretation of data, drafting and revising of the article, giving final approval to the version to be published.

## Ethical statement

This contact tracing study is embedded in the mandatory routine surveillance conducted by the Public Health Department, as legally mandated by the German Infectious Diseases Law (IfSG) and has been approved by the Hamburg Commissioner for Data Protection. Approval by an ethics committee was therefore not required.

## CRediT authorship contribution statement

**Roland Diel:** Writing – review & editing, Writing – original draft, Methodology, Formal analysis, Data curation, Conceptualization. **Matthias Gröschel:** Writing – review & editing, Methodology, Formal analysis. **Albert Nienhaus:** Writing – review & editing, Methodology, Formal analysis.

## Declaration of competing interest

The authors declare that they have no known competing financial interests or personal relationships that could have appeared to influence the work reported in this paper.
